# Factor analysis of the biochemical markers related to liver cirrhosis

**DOI:** 10.12669/pjms.315.8025

**Published:** 2015

**Authors:** Hyun-Jin Kim, Hae-Kag Lee, Jae-Hwan Cho

**Affiliations:** 1 Hyun-Jin Kim, Department of Radiology, Asan Medical Center, Department of International Radiological Science, Hallym University of Graduate Studies, Seoul, Republic of Korea; 2 Hae-Kag Lee, Department of Computer Science and Engineering, Soonchunhyang University, Asan, Republic of Korea; 3 Jae-Hwan Cho, Department of International Radiological Science, Hallym University of Graduate Studies, Seoul, Republic of Korea

**Keywords:** Correlations, Biochemical study, Liver cirrhosis

## Abstract

**Objectives:**

The purpose of this study was to find the correlations between biochemical study and liver cirrhosis.

**Methods:**

The patients had liver biopsy to check the degree of their liver fibrosis, from August 2013 to August 2014 at the current medical center. In order to find the etiology of hepatitis, a research was done on gender, age, weight, and biochemical study through the investigation of subjects’ medical record and medical history. For biochemical study, we examined hemoglobin, platelets, albumin, aspartate aminotransferase (AST), alanine aminotransferase (ALT), total bilirubin, gamma-glutamyl transferase (GGT), prothrombin time (PT), and international normalised ratio (INR). We also analyzed the factors that are related to liver cirrhosis.

**Results:**

As a result, the patients at liver cirrhosis F≥2 stage showed 0.973, which is higher than the patients at FO stage with 0.943. F≥2 stage of hemoglobin was 0.544, which is lower than FO stage of hemoglobin with 0.817. Platelet count in F≥2 stage was 0.417, which is higher than FO stage with 0.074. For Albumin, F≥2 stage was 0.155 when F0 stage was 0.135. ATS’s F≥2 stage was 0.665, which is 6 times higher than FO stage with 0.100. Moreover, in the case of GGT, F≥2 stage was higher with 0.492 than FO stage with 0.078.

**Conclusions:**

In conclusion, it was confirmed that there is an increase in liver cirrhosis in the following general characteristics and biochemical factors: increase of age, increase of GGT, decrease of albumin, increase of the total bilirubin, and growth of INR (International Normalized Ratio).

## INTRODUCTION

Liver cirrhosis is a disease when a normal cell is replaced with scar tissue, and the human body becomes disturbed in the performance of essential functions. In liver cirrhosis, liver cells are destroyed without being regenerated, and they become solid and are decreased.^1^-^4^

Liver cirrhosis is a liver disease and one of the liver cancers. It is also one of the main chronic diseases or the main cause of liver disease in Korea. Compared to the Westerners, Koreans are more likely to get infected by hepatitis, there is a higher repetition rate of liver cirrhosis.^5^,^6^ Since the rate of drinking alcoholic beverages is increasing^7^, the occurrence of chronic liver disease is becoming worse. Liver cirrhosis mostly appears when chronic hepatitis due to hepatitis virus is not well treated. Because of the hepatitis and other factors, normal liver cells are destroyed and they decrease in numbers; this changes the normal structure of liver and disturbs the liver functions.^8^,^9^ There are no specific symptoms of liver cirrhosis at early stages. However, as more liver cells are destroyed, the production of proteins that manage body fluid congestion and blood coagulation, and the metabolism of bilirubin are decreased. For these reasons, there are various complications. Deaths frequently occur due to the serious damage in the liver due to various complications. Therefore, it is very important to prevent varicose vein, seroperitoneum and edema, hepatic encephalopathy, and liver cancer to treat liver cirrhosis.^10^

The methods used for examination and diagnosis of liver cirrhosis are imaging diagnosis and tissue pathology diagnosis; and computed tomography (CT), magnetic resonance imaging (MRI), and Sonography are being widely used.

CT and MRI are costly, and they have side-effects due to the use of contrast media. On the other hand, Sonography is preferred for imaging diagnosis of liver cirrhosis because of its safety, repeatability, and cost.^11^ In terms of variables related to the symptoms of liver cirrhosis, sociological variables such as age and gender have been reported.^12^ In most of the studies, only the correlation of child-pugh score (sometimes the Child-Turcotte-Pugh score), etiology of liver cirrhosis, and physiological variables have been confirmed.^13^,^14^ However, there has been no clear report about factors related to blood count and biochemical test. Therefore, this study was conducted to find the correlation between biochemical test and liver cirrhosis.

## METHODS

A retrospective analysis was conducted on 304 patients who underwent ultrasound test before and after the liver biopsy. They were selected among the patients who received liver biopsy to check the degree of liver fibrosis, from August 2013 to August 2014 at the current hospital. In order to find the etiology of hepatitis, a research was conducted on gender, age, weight, and biochemical study by investigating the subjects’ medical record and medical history. In serological study, the patients with chronic hepatitis B virus had positive HBsAg for over-6-month, and the patients with chronic hepatitis C virus had positive anti-HCV and hepatitis C virus (HCV) RNA for over-6-month. The patients with alcoholic hepatitis were also included in this study. Other types of hepatitis caused by the unidentified factors were excluded.

There were a total of 221 subjects which included 144 patients with hepatitis B virus (HBV), 71 patients with HCV, and 6 patients with alcoholic hepatitis. Among all of the subjects, there were 125 men (56.6%) and 96 women (44.3%), and the average age of the subjects was 54.79±11.46. The ultrasound guided percutaneous liver biopsy was performed with a fixed sample with formalin solution. The sample was fixed with formalin solution and treated with paraffin. Then, masson-trichrome staining was done to have a precise understanding of the liver fibrosis and the staining with hematoxylin-eosin was done after sectioning into a thickness of 5μm. According to the guidelines of Korean Society of Pathologies’ digestive system of pathology, the system and labeling of hepatitis are categorized into the followings: no fibrosis (F0), portal fibrosis (F1), periportal fibrosis without liver cirrhosis (F2), septal fibrosis (F3), and liver cirrhosis (cirrhosis, F4).^15^ In addition, F≥2 stage was analyzed by dividing into significant fibrosis, and F≥3 stage by dividing into advanced fibrosis.

The following 5 items were used for evaluating the individual index: gender, age, height, weight, and degree of obesity. In order to process biochemical index, subjects were instructed to fast for at least 12 hours, and blood and urine tests were given. With the outcomes, we examined 9 factors including the followings: hemoglobin, platelets, albumin, AST, ALT, total bilirubin, GGT, PT, and INR. In order to analyze the factors related to liver cirrhosis, we performed Pearson Correlation coefficient and linear regression analysis. In addition, we conducted logistic regression analysis on the related factors, Pearson’s Chi-square test by subject’s general characteristic, and Pearson’s Chi-square test by hematological index.

## RESULTS

The results from the frequency analysis of liver cirrhosis by subjects were as follows: 19 patients in F=0 stage (8.6%), which indicates normal; 29 patients in F≥1 stage (13.1%); 50 patients in F≥2 stage (22.6%); 51 patients in F≥3 stage (23.1%); and 72 patients in F≥4 stage (32.6%) (Table-I). In order to identify the characteristic correlation between liver cirrhosis and variables, we conducted simple correlation analysis by variables. As a result, we found the correlation in variables as follows: age was positive 0.260, Hemoglobin (g/dL) was negative 0.152, Platelet (10³/UL) was negative 0.287, AST (IU/L) was positive 0.209, Total bilirubin (mg/dL) was positive 0.194, GGT (IU/L) was positive 0.175, prothrombin time(%) was negative 0.178, and INR was positive 0.352 (p<0.05) (Table-II). By conducting linear regression analysis on liver cirrhosis related factors, we were able to compute constant, standard error, and significant probability of each variable and liver biopsy result. There was statistical significance between the result from liver biopsy of liver cirrhosis and the value of age, platelet (10³/UL), INR in 0.000, Hemoglobin (g/dL) in 0.024, Total bilirubin (mg/dL) in 0.004, and prothrombin time(%) in 0.008 (p<0.05) (Table-III). For the precise correlation analysis, simple correlation analysis was performed, and then the multivariate analysis was conducted through logistic regression analysis by using related variables to obtain the odds ratio [Exp(B)]. The odds ratio [Exp(B)] in age for liver cirrhosis patients in F≥2 stage was 0.973 which is higher than the patients in FO stage that was 0.943. For Hemoglobin, F≥2 stage was 0.544 which is lower than F0 that was 0.817. For Platelet count, F≥2 stage was 0.417 which is higher than FO stage that was 0.074. Also, Albumin’s F≥2 stage was 0.155 which is higher than F0 stage that was 0.135. AST’s F≥2 stage was 0.665 which is over 6 times higher than FO stage that was 0.100. Gamma GT’s F≥2 stage was 0.492 which is higher than FO stage that was 0.078 (Table-IV).

**Table-I:** The results from the frequency analysis of liver cirrhosis.

Division	Liver cirrhosis	Frequency	%
Biopsy	F=0	19	8.6
	F≥1	29	13.1
	F≥2	50	22.6
	F≥3	51	23.1
	F=4	72	32.6

**Table-II:** The characteristic correlation between liver cirrhosis and variables.

Variable	Liver cirrhosis	Variable	Liver cirrhosis
Sex	-0.075	^1^AST	0.209
Years	0.260	^2^ALT	0.073
Height	-0.004	Total bilirubin	0.194
Weight	0.000	^3^GGT	0.175
Obesity	0.015	^4^PT	-0.178
Pathogenesis	0.080	^5^INR	0.352
Hemoglobin	-0.152		
Platelets	-0.287		
Albumin	-0.230		

1 Aspartate aminotransferase,

2 Alanine aminotransferase,

3 Gamma-glutamyl transferase,

4 Prothrombin time,

5 International normalised ratio

**Table-III:** Linear regression analysis on liver cirrhosis related factors.

Variable	B	SE
Age	2.299	0.577
Hemoglobin	-0.230	0.101
Platelets	-19.271	4.348
Albumin	-0.173	0.039
^1^AST	6.540	2.073
total bilirubin	0.414	0.141
^2^GGT	10.423	3.963
^3^PT	-5.263	1.967
^4^INR	0.063	0.011

1 Aspartate aminotransferase,

2 Gamma-glutamyl transferase,

3 Prothrombin time,

4 International normalised ratio

**Table-IV:** Logistic regression analysis on liver cirrhosis related factors.

Variable	Biopsy Classification	B	SE	Exp(B)
Age	0	-0.059	0.023	0.943
	1	-0.069	0.020	0.933
	2	-0.027	0.016	0.973
	3	-0.023	0.016	0.977
Hemoglobin	0	-0.203	0.532	0.817
	1	-1.496	0.589	0.224
	2	-0.608	0.395	0.544
	3	-1.075	0.426	0.341
Platelets	0	-2.606	0.678	0.074
	1	-1.582	0.464	0.206
	2	-0.876	0.376	0.417
	3	-0.132	0.382	0.876
Albumin	0	-2.003	1.060	0.135
	1	-2.445	1.050	0.087
	2	-1.864	0.649	0.155
	3	-1.332	0.538	0.264
AST	0	-2.307	0.784	0.100
	1	-0.966	0.466	0.381
	2	-0.408	0.370	0.665
	3	-0.364	0.368	0.695
Total bilirubin	0	-2.857	1.066	0.467
	1	-0.807	0.601	0.446
	2	-1.171	0.542	0.310
	3	-0.385	0.443	0.680
GGT	0	-2.554	1.055	0.078
	1	-0.629	0.479	0.533
	2	-0.709	0.401	0.492
	3	-1.204	0.438	0.300
PT	0	-0.693	0.573	0.500
	1	-0.462	0.467	0.630
	2	-0.153	0.377	0.858
	3	-0.102	0.373	0.903

^1^Aspartate aminotransferase, ^2^Gamma-glutamyl transferase, ^3^Prothrombin time, ^4^International normalised ratio

## DISCUSSION

In this study, we analyzed the correlations between liver cirrhosis and general characteristic, as well as liver cirrhosis and biochemical examination of blood. Some factors showed a negative correlation. Coco and others have reported that the counts of ALT and AST were elevated in the patients with chronic viral hepatitis.^16^,^17^ According to other studies, the changes of total bilirubin count had an impact on liver cirrhosis.^18^ In our study, we looked for other factors that affect liver stiffness from these patients with chronic liver disease. As a result of analyzing how individual characteristic index and biochemical index of blood are related to liver cirrhosis, the followings were shown: growth of elderly, increase of GGT, decrease of albumin, increase of total bilirubin, and decrease of Gamma GT. Although there was an increase of total bilirubin in other studies, there was no report on the changes of ALT value, which differentiate this study from other studies.

A decrease of albumin can cause tissue edema, which results in more serious liver disease and liver cirrhosis. On the basis of the result of this study, overall clinical factors should be considered through medical history, serological examination, and imaging examination of the patients with chronic liver disease, who have no signs of acute exacerbation or superinfection of other acute hepatitis. Especially, when there are growth of elderly, increase of GGT, decrease of albumin and increase of total bilirubin, we can measure liver cirrhosis because these are the signs of increased liver cirrhosis. If there were growth of elderly, increase of gamma GT, decrease of albumin, increase of total bilirubin and increase of INR, it indicates that the liver cirrhosis is being enhanced. Through this study, we assumed that there has to be a massive retrospective study about the direct cause of liver cirrhosis, since it has not been discovered yet.

## CONCLUSIONS

In conclusion, it was confirmed that there is an increase in liver cirrhosis in the following general characteristics and biochemical factors: increase of age, increase of GGT, decrease of albumin, increase of the total bilirubin, and growth of INR (International Normalized Ratio).
